# Clinical nomogram prediction model to assess the risk of prolonged ICU length of stay in patients with diabetic ketoacidosis: a retrospective analysis based on the MIMIC-IV database

**DOI:** 10.1186/s12871-024-02467-z

**Published:** 2024-02-29

**Authors:** Jincun Shi, Fujin Chen, Kaihui Zheng, Tong Su, Xiaobo Wang, Jianhua Wu, Bukao Ni, Yujie Pan

**Affiliations:** Department of Critical Care Medicine, Wenzhou Central Hospital, Wenzhou, Zhejiang 325000 China

**Keywords:** Diabetic ketoacidosis, Intensive care unit, Length of stay, Nomogram prediction model, MIMIC-IV database

## Abstract

**Background:**

The duration of hospitalization, especially in the intensive care unit (ICU), for patients with diabetic ketoacidosis (DKA) is influenced by patient prognosis and treatment costs. Reducing ICU length of stay (LOS) in patients with DKA is crucial for optimising healthcare resources utilization. This study aimed to establish a nomogram prediction model to identify the risk factors influencing prolonged LOS in ICU-managed patients with DKA, which will serve as a basis for clinical treatment, healthcare safety, and quality management research.

**Methods:**

In this single-centre retrospective cohort study, we performed a retrospective analysis using relevant data extracted from the Medical Information Mart for Intensive Care IV (MIMIC-IV) database. Clinical data from 669 patients with DKA requiring ICU treatment were included. Variables were selected using the Least Absolute Shrinkage and Selection Operator (LASSO) binary logistic regression model. Subsequently, the selected variables were subjected to a multifactorial logistic regression analysis to determine independent risk factors for prolonged ICU LOS in patients with DKA. A nomogram prediction model was constructed based on the identified predictors. The multivariate variables included in this nomogram prediction model were the Oxford acute severity of illness score (OASIS), Glasgow coma scale (GCS), acute kidney injury (AKI) stage, vasoactive agents, and myocardial infarction.

**Results:**

The prediction model had a high predictive efficacy, with an area under the curve value of 0.870 (95% confidence interval [CI], 0.831–0.908) in the training cohort and 0.858 (95% CI, 0.799–0.916) in the validation cohort. A highly accurate predictive model was depicted in both cohorts using the Hosmer–Lemeshow (H-L) test and calibration plots.

**Conclusion:**

The nomogram prediction model proposed in this study has a high clinical application value for predicting prolonged ICU LOS in patients with DKA. This model can help clinicians identify patients with DKA at risk of prolonged ICU LOS, thereby enhancing prompt intervention and improving prognosis.

**Supplementary Information:**

The online version contains supplementary material available at 10.1186/s12871-024-02467-z.

## Background

Diabetic ketoacidosis (DKA) occurs when insulin secretion is insufficient to inhibit the production of blood ketone bodies. It is the most common acute life-threatening complication in patients with diabetes mellitus and is one of the leading causes of death in this patient population [1, 2]. Although the mortality rate of patients with DKA is decreasing, the hospitalisation rate remains high [3, 4] especially as the length of stay (LOS) for patients with DKA requiring further treatment in the intensive care unit (ICU) continues to rise. This trend places a great burden on healthcare resources worldwide. ICU costs account for a large part of the total cost of hospitalisation, posing a great challenge to the global economy [5, 6].

The length of hospitalisation in patients with DKA may be influenced by their prognosis. Ata et al. [7] showed that patients with DKA with longer hospitalisation periods are at a higher risk of comorbidities, leading to a poor prognosis, further increasing the economic burden, and creating a vicious circle. Patients with DKA treated in general wards have been reported to exhibit no significant difference in mortality rate but had lower treatment costs compared to patients with DKA admitted to the ICU [8, 9]. Therefore, the LOS of patients with DKA, especially in the ICU, is closely related to patient prognosis and treatment cost.

Previous studies have shown that several factors influence LOS in patients with DKA. However, a highly sensitive and specific predictive model to assess ICU LOS in patients with DKA is lacking [7, 10]. Therefore, this study aimed to develop a nomogram prediction model to identify the risk factors influencing prolonged ICU LOS in patients with DKA. This model may serve as a basis for clinical treatment, healthcare safety, and quality management studies.

## Methods

### Study design and data source

We performed a retrospective analysis using all relevant data extracted from the MIMIC-IV database [11]. This database comprised patient-related data collected in the ICUs of the Beth Israel Deaconess Medical Centre between 2008 and 2019. This public-access database is supported by the Department of Medicine at the Beth Israel Deaconess Medical Centre and the Computational Physiology Laboratory at Massachusetts Institute of Technology (MIT) and is freely accessible to any qualified PhysioNet user. CFJ received a certificate (No: 43,529,529) and permission to use the MIMIC-IV database after completing the web-based course. The Institutional Review Boards of MIT (Cambridge, MA, USA) and the Beth Israel Deaconess Medical Centre (Boston, MA, USA) approved the data collection and use of MIMIC-IV for research purposes and granted a waiver of informed consent.

### Participant selection criteria

In this study, all patients admitted to the ICU who met the diagnostic criteria for DKA were included following the ICD-9/10 diagnostic codes in the database [1]. The diagnostic criteria for DKA were as follows: (i) Glucose > 13.8 mmol/L, (ii) positive urine or serum ketones positive or β-hydroxybutyrate > 3 mmol/L, and (iii) arterial or venous pH < 7.3, bicarbonate < 18 mmol/L and anion gap > 10–12 mmol/L [1].

**Fig. 1:**
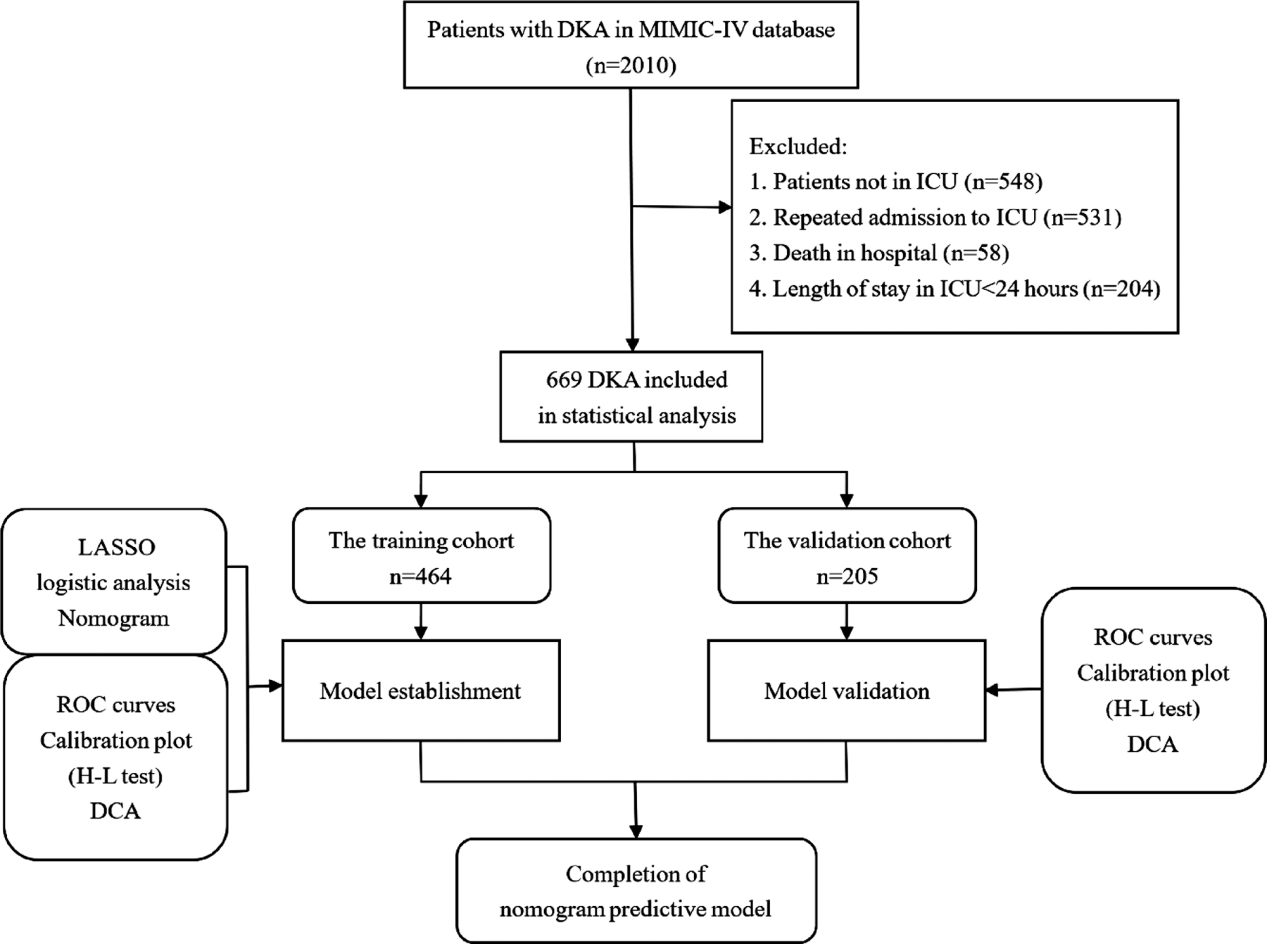
The flowchart of the study

Patients with repeat ICU admissions, in-hospital deaths, and those admitted to the ICU for less than 24 h were excluded. A total of 669 patients with DKA were randomly assigned in a 7:3 ratio to a training cohort (*n* = 464) for nomogram model development and a validation cohort (*n* = 205) for internal validation of the nomogram model’s performance. The study population enrolment flowchart is presented in Fig. 1.

### Data extraction and definition of terms

Several variables were extracted from the database, including patient demographics, vital signs, comorbidities, laboratory indicators, scoring systems, and medical interventions. All data were collected within 24 h of ICU admission. Considering that several variables were measured multiple times, the worst values of laboratory variables recorded within 24 h of ICU admission were used for analysis and included in the predictive model.

ICU LOS was defined as the period from day 1 of ICU admission to the day before transfer from the ICU. Prolonged ICU LOS was defined as an ICU LOS ≥ 75th percentile (i.e., ≥ 75 h) of ICU LOS for all patients enrolled in this study. The cohorts were divided into the normal and prolonged groups according to whether the ICU LOS was ≥ 75 h.

### Statistical analysis

Statistical analyses were performed using R version 4.3.1 (R Foundation for Statistical Computing, Vienna, Austria). Normally distributed measures were expressed as mean ± standard deviation, non-normally distributed measures as medians and quartiles, and count data as frequencies and percentages. The unpaired *t*-test was used to compare group values that conformed to a normal distribution, the Mann–Whitney *U* test was used to compare group values that did not conform to a normal distribution, and the chi-square test was used to compare categorical variables. Parameters with more than 20% missing values were excluded from the analysis. The missing values for all extracted variables are listed in Supplementary Table 1. Values missing for other parameters were filled in using multiple imputations with the ‘mice’ package of R software. First, the R ‘caret’ package was used to randomly divide the 669 patients with DKA into a training set with 464 participants and a validation set with 205 participants for external validation, conforming to the theoretical ratio of 7:3. Least absolute shrinkage and selection operator (LASSO), a shrinkage and variable selection method for linear regression models were performed using the ‘glmnet’ package. The ‘rms’ package was then used to develop the nomogram diagram based on a multivariable logistic regression analysis. The analysis was used to construct a predictive model by introducing the features selected in the LASSO regression model. The receiver operating characteristic (ROC) curves were plotted, and the area under the curve (AUC) was calculated using the ‘pROC’ package. These curves and calculations were used to assess the discriminatory ability of nomograms. We used the rms package to draw and calculate the calibration curves via the Hosmer–Lemeshow test. These curves were employed to evaluate the calibration of nomograms.

For the assessment of clinical practicability based on net benefit under various threshold probabilities, decision curve analysis (DCA) was conducted using the ‘rmda’ package. A P-value of < 0.050 was considered statistically significant.

## Results

### Patient clinical characteristics

A total of 669 patients with DKA were included in the analysis, of which 464 were in the training cohort and 205 in the validation cohort. The training and validation cohorts comprised 236 and 128 male patients, respectively. In both cohorts, the DKA stage was predominantly mild (59.3% in the training group and 70.2% in the validation group), and type 1 diabetes mellitus (T1DM) was the predominant diagnosis. The training cohort showed slightly higher chloride levels than the validation cohort. The training cohort had slightly lower bicarbonate, potential of hydrogen, and red blood cell distribution widths than the validation cohort. There were no significant differences between the training and validation cohort patients regarding the other variables (*P* > 0.050). These results justify the use of training and validation cohorts. Detailed clinical characteristics of the patients are listed in Table 1.

**Table 1:** Summary statistics of patient clinical characteristics

Variables	Training cohort			Validation cohort					*P*
	Total (*n* = 464)	Normal (*n* = 334)	Prolonged (*n* = 120)	Total (*n* = 205)		Normal (*n* = 149)		Prolonged (*n* = 56)	
Age, (year)	49.0 (35.0,62.0)	47.0 (31.8,60.0)	54.0 (44.8,65.0)	50.0 (36.0,62.0)		48.0 (34.0,61.0)		56.5 (46.8,65.2)	0.342
BMI, (kg/m^2^)	26.6 (22.8,31.4)	26.5 (23.2,30.9)	27.3 (22.5,32.2)	27.5 (23.6,32.3)		27.4 (23.4,32.0)		28.2 (24.0,32.4)	0.119
Male, n (%)	236 (50.9%)	161 (46.8%)	75 (62.5%)	128 (62.4%)		96 (64.4%)		32 (57.1%)	0.007
**Ethnicity, n (%)**									0.309
Caucasian	266 (57.3%)	195 (56.7%)	71 (59.2%)	128 (62.4%)		96 (64.4%)		32 (57.1%)	
African American	108 (23.3%)	92 (26.7%)	16 (13.3%)	44 (21.5%)		33 (22.1%)		11 (19.6%)	
Latin American	15 (3.23%)	21 (6.10%)	5 (4.17%)	3 (1.46%)		4 (2.68%)		2 (3.57%)	
Asian	26 (5.60%)	10 (2.91%)	5 (4.17%)	6 (2.93%)		3 (2.01%)		0 (0.00%)	
Other	49 (10.6%)	26 (7.56%)	23 (19.2%)	24 (11.7%)		13 (8.72%)		11 (19.6%)	
**DM type, n (%)**									0.003
T1DM	265 (57.1%)	205 (59.6%)	60 (50.0%)	97 (47.3%)		82 (55.0%)		15 (26.8%)	
T2DM	88 (19.0%)	61 (17.7%)	27 (22.5%)	63 (30.7%)		45 (30.2%)		18 (32.1%)	
Unknown	111 (23.9%)	78 (22.7%)	33 (27.5%)	45 (22.0%)		22 (14.8%)		23 (41.1%)	
**DKA stage**									0.016
Mild	275 (59.3%)	211 (61.3%)	64 (53.3%)	144 (70.2%)		108 (72.5%)		36 (64.3%)	
Moderate	106 (22.8%)	68 (19.8%)	38 (31.7%)	39 (19.0%)		25 (16.8%)		14 (25.0%)	
Severe	83 (17.9%)	65 (18.9%)	18 (15.0%)	22 (10.7%)		16 (10.7%)		6 (10.7%)	
Emergency admission, n (%)	304 (65.5%)	226 (65.7%)	78 (65.0%)	137 (66.8%)		107 (71.8%)		30 (53.6%)	0.809
**Medical History, n (%)**									
Congestive heart failure	83 (17.9%)	50 (14.5%)	33 (27.5%)	37 (18.0%)		19 (12.8%)		18 (32.1%)	1.000
Myocardial infarct	78 (16.8%)	37 (10.8%)	41 (34.2%)	36 (17.6%)		22 (14.8%)		14 (25.0%)	0.899
Peripheral vascular disease	36 (7.76%)	23 (6.69%)	13 (10.8%)	17 (8.29%)		11 (7.38%)		6 (10.7%)	0.936
Cerebrovascular disease	30 (6.47%)	12 (3.49%)	18 (15.0%)	14 (6.83%)		2 (1.34%)		12 (21.4%)	0.995
Chronic pulmonary disease	60 (12.9%)	41 (11.9%)	19 (15.8%)	31 (15.1%)		16 (10.7%)		15 (26.8%)	0.522
Renal disease	108 (23.3%)	74 (21.5%)	34 (28.3%)	63 (30.7%)		42 (28.2%)		21 (37.5%)	0.052
Malignant cancer	16 (3.45%)	12 (3.49%)	4 (3.33%)	14 (6.83%)		11 (7.38%)		3 (5.36%)	0.081
RR, (breaths/min)	20.0 (16.0,24.0)	20.0 (16.0,23.2)	20.5 (17.8,26.2)	20.0 (16.0,24.0)		19.0 (16.0,23.0)		20.0 (18.0,24.0)	0.460
HR, (beats/min)	100 (88.0,114)	101 (88.0,113)	99.0 (85.8,115)	98.0 (89.0,108)		98.0 (87.0,108)		100 (92.0,109)	0.242
Temperature, (°C)	36.8 (36.6,37.1)	36.8 (36.6,37.1)	36.8 (36.4,37.1)	36.9 (36.6,37.2)		36.8 (36.6,37.1)		37.1 (36.7,37.4)	0.199
MAP, (mmHg)	85.0 (73.0,96.0)	87.0 (74.0,96.0)	81.5 (70.8,97.0)	88.0 (76.0,99.0)		89.0 (77.0,100)		86.0 (70.0,99.0)	0.097
UO, (ml)	2238 (1324,3550)	2335 (1448,3700)	1870 (939,2778)	2165 (1358,3010)		2250 (1385,3390)		1802 (1184,2540)	0.137
GCS	15.0 (14.0,15.0)	15.0 (14.0,15.0)	13.0 (9.00,14.0)	14.0 (13.0,15.0)		15.0 (14.0,15.0)		13.0 (12.0,14.0)	0.323
SOFA	3.00 (1.00,5.00)	2.00 (1.00,3.25)	6.00 (4.00,10.0)	3.00 (2.00,5.00)		3.00 (1.00,4.00)		7.00 (3.00,9.00)	0.152
LODS	3.00 (1.00,5.00)	3.00 (1.00,4.00)	6.00 (4.00,9.00)	4.00 (2.00,6.00)		3.00 (1.00,5.00)		6.00 (3.75,8.25)	0.206
OASIS	26.0 (21.8,33.0)	24.0 (20.0,29.0)	34.0 (29.0,41.0)	26.0 (21.0,33.0)		24.0 (20.0,29.0)		35.5 (27.5,42.2)	0.812
SAPSII	28.0 (20.0,38.0)	25.0 (18.0,34.0)	38.0 (30.0,47.0)	28.0 (21.0,40.0)		24.0 (19.0,34.0)		38.0 (27.0,50.0)	0.631
APSIII	46.0 (36.0,59.0)	44.0 (34.0,53.2)	58.0 (43.0,72.2)	46.0 (37.0,57.0)		43.0 (34.0,53.0)		55.0 (47.0,73.2)	0.884
CCI	4.00 (2.00,7.00)	3.00 (2.00,6.00)	5.00 (3.00,8.00)	5.00 (2.00,7.00)		4.00 (2.00,7.00)		7.00 (4.00,8.00)	0.096
WBC, (10^9^/L)	14.0 (10.1,18.8)	13.7 (9.58,17.9)	14.8 (12.0,20.2)	13.3 (9.20,18.2)		12.9 (9.00,18.2)		14.6 (10.9,18.5)	0.188
Platelets, (10^9^/L)	218 (169,274)	222 (176,275)	208 (146,273)	215 (160,264)		215 (167,269)		198 (139,261)	0.363
Hematocrit, (%)	32.1 (28.4,36.1)	32.5 (28.9,36.3)	31.6 (26.7,35.8)	32.0 (27.2,36.7)		32.7 (27.8,37.2)		30.8 (26.1,35.6)	0.833
RDW, (%)	13.6 (12.8,14.5)	13.5 (12.7,14.5)	13.8 (13.1,14.5)	13.8 (13.0,15.3)		13.7 (12.9,14.7)		14.1 (13.2,16.1)	0.017
Hemoglobin, (g/dL)	10.8 (9.30,12.1)	11.0 (9.40,12.3)	10.4 (8.78,11.8)	10.8 (8.90,12.3)		11.1 (9.30,12.5)		9.95 (8.40,11.9)	0.981
AG, (mmol/L)	26.0 (21.0,33.0)	26.0 (22.0,33.0)	27.0 (21.0,33.0)	25.0 (21.0,30.0)		26.0 (22.0,31.0)		23.0 (20.0,27.0)	0.198
Lactate, (mmol/L)	2.20 (1.50,3.40)	2.10 (1.50,3.20)	2.60 (1.67,4.00)	2.20 (1.60,3.60)		2.10 (1.60,3.20)		2.35 (1.60,4.40)	0.990
Bicarbonate, (mmol/L)	13.0 (8.00,17.0)	13.0 (8.00,17.0)	13.0 (8.00,18.0)	14.0 (10.0,18.0)		13.0 (10.0,18.0)		15.0 (11.0,19.0)	0.010
Chloride, (mmol/L)	109 (105,114)	109 (105,114)	109 (105,114)	108 (104,112)		108 (104,112)		108 (106,114)	0.041
Sodium min, (mmol/L)	133 (130,137)	133 (130,136)	134 (130,139)	133 (129,136)		133 (128,135)		134 (129,141)	0.305
Sodium max, (mmol/L)	140 (137,143)	140 (138,143)	142 (137,145)	139 (137,143)		139 (137,142)		142 (137,144)	0.065
Potassium min, (mmol/L)	3.60 (3.20,3.90)	3.60 (3.27,3.90)	3.50 (3.17,4.00)	3.70 (3.30,4.00)		3.70 (3.30,4.00)		3.50 (3.30,3.80)	0.141
Potassium max, (mmol/L)	5.00 (4.40,5.70)	5.00 (4.40,5.70)	5.00 (4.40,5.62)	5.10 (4.40,6.00)		5.10 (4.50,6.00)		4.90 (4.30,5.88)	0.254
Calcium, (mg/dL)	7.80 (7.40,8.33)	7.85 (7.40,8.30)	7.80 (7.20,8.40)	7.90 (7.50,8.30)		8.00 (7.60,8.40)		7.70 (7.20,8.20)	0.238
Glucose min, (mg/dL)	95.0 (73.0,120)	94.0 (72.0,118)	99.5 (78.0,129)	96.0 (76.0,122)		93.0 (76.0,125)		99.0 (76.0,120)	0.726
Glucose max, (mg/dL)	360 (293,460)	352 (290,446)	386 (324,492)	373 (302,468)		364 (297,453)		394 (322,484)	0.507
Creatinine, (mg/dL)	1.40 (1.00,2.10)	1.30 (0.97,1.90)	1.80 (1.20,3.05)	1.50 (1.00,2.30)		1.30 (1.00,2.10)		1.75 (1.30,2.85)	0.297
BUN, (mg/dL)	26.0 (16.0,45.0)	25.0 (14.0,39.0)	38.5 (19.8,60.0)	31.0 (18.0,51.0)		27.0 (17.0,46.0)		39.0 (22.8,60.5)	0.052
POP, (mOsm/kg)	294 (287,302)	294 (287,302)	297 (286,308)	293 (286,301)		292 (286,300)		295 (290,303)	0.194
PH	7.27 (7.15,7.33)	7.27 (7.16,7.33)	7.24 (7.13,7.33)	7.29 (7.20,7.35)		7.29 (7.21,7.36)		7.28 (7.20,7.34)	0.004
**Complication, n (%)**									
Suspected infection	259 (55.8%)	162 (47.1%)	97 (80.8%)	117 (57.1%)		66 (44.3%)		51 (91.1%)	0.828
HHS	154 (33.2%)	106 (30.8%)	48 (40.0%)	57 (27.8%)		37 (24.8%)		20 (35.7%)	0.197
AKI									0.658
Stage 0-I	342 (73.7%)	294 (85.5%)	48 (40.0%)	147 (71.7%)		127 (85.2%)		20 (35.7%)	
Stage II-III	122 (26.3%)	50 (14.5%)	72 (60.0%)	58 (28.3%)		22 (14.8%)		36 (64.3%)	
**Treatment, n (%)**									
CRRT	12 (2.59%)	2 (0.58%)	10 (8.33%)	3 (1.46%)		1 (0.67%)		2 (3.57%)	0.572
Mechanical ventilation	64 (13.8%)	16 (4.65%)	48 (40.0%)	32 (15.6%)		8 (5.37%)		24 (42.9%)	0.618
Vasoactive agent	64 (13.8%)	16 (4.65%)	48 (40.0%)	33 (16.1%)		7 (4.70%)		26 (46.4%)	0.508
Heparin	365 (78.7%)	280 (81.4%)	85 (70.8%)	160 (78.0%)		120 (80.5%)		40 (71.4%)	0.939
Insulin, (U)	71.6 (33.8,118)	66.9 (32.2,111)	84.7 (38.7,181)	67.1 (33.2,115)		62.3 (33.0,98.5)		101 (57.0,169)	0.751
Infusion volume, (ml)	6017 (4053,8369)	5771 (3990,7869)	6792 (4256,10006)		5856 (3925,8414)		5663 (3762,8118)	6549 (4323,8900)	0.939

*Abbreviations* BMI Body mass index, DM Diabetic mellitus, T1DM Type 1 diabetic mellitus, T2DM Type 2 diabetic mellitus, RR Respiratory rate, HR Heart rate, MAP Mean arterial pressure, UO Urine volume, GCS Glasgow coma scale, SOFA Sequential organ failure assessment, LODS Logistic organ dysfunction system, OASIS Oxford acute severity of illness score, SAPSII Simplified acute physiology score II, APSIII Acute physiology score, CCI Charlson comorbidity index, WBC White blood cell, RDW Red blood cell distribution width, AG Anion gap, POP Plasma osmotic pressure, PH Potential hydrogen, BUN Blood urea nitrogen, HHS Hyperosmolar hyperglycemia state, AKI Acute kidney injury, CRRT Continuous renal replacement therapy

### Variable selection

**Fig. 2:**
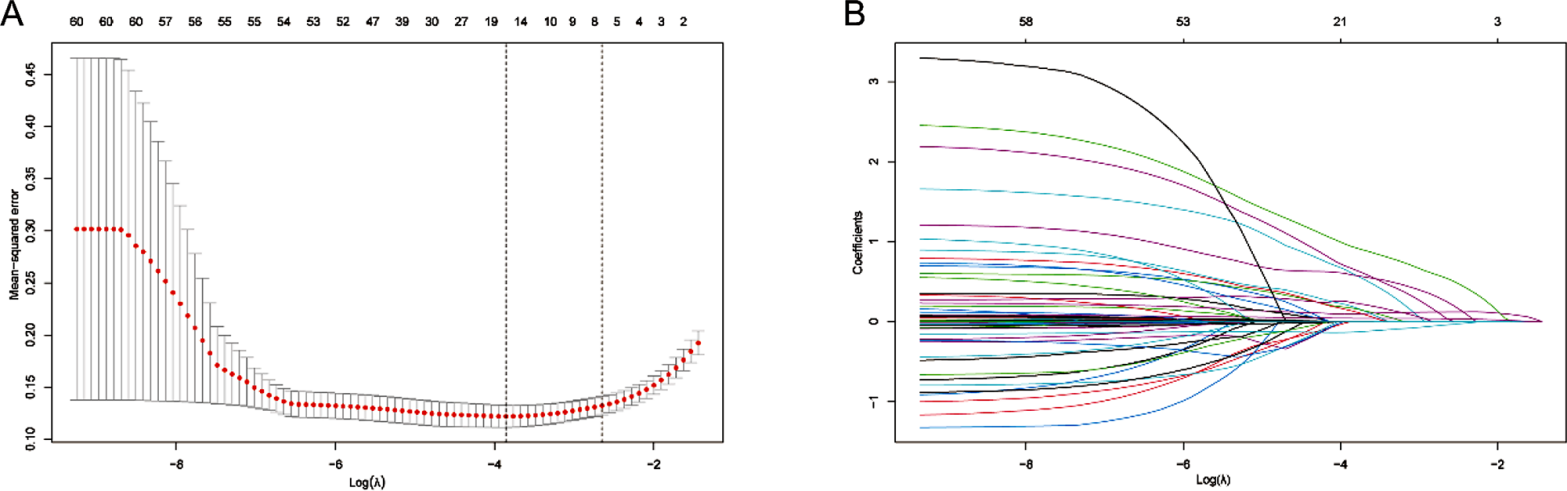
Variable selection by the LASSO binary logistic regression model. **A**. The process of selecting the most suitable λ (0.071) in the LASSO model by means of 10-fold cross-validation. **B**. Six variables with nonzero coefficients were selected by deriving the optimal lambda

Based on the demographics, vital signs, medical history, laboratory parameters, scoring system, and patients’ treatments in the training cohort, six predictor variables with non-zero coefficients were identified out of the initial 60 variables using LASSO regression analysis (Fig. 2). Vertical lines were plotted at the minimum value of λ (λ = 0.021) and the value of 1 standard error (SE) from the minimum value (λ = 0.071). At the point where log(λ) = -1.150, six non-zero coefficients were identified as the most appropriate predictor variables in the LASSO regression model. The predictor variables included Oxford acute severity of illness score (OASIS), Glasgow coma scale (GCS), Acute kidney injury (AKI) stage, vasoactive agents, and myocardial infarction.

### Construction of nomogram prediction model

**Fig. 3:**
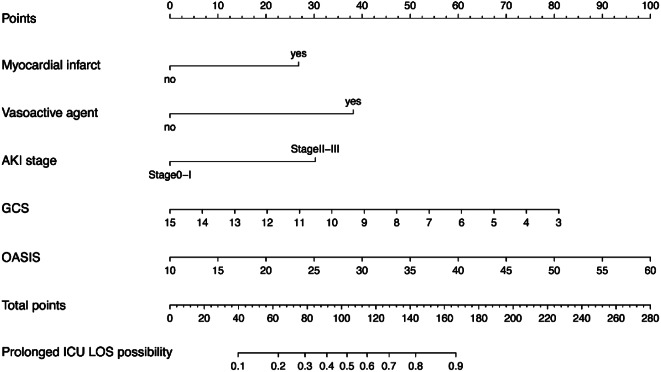
Nomogram for predicting prolonged ICU LOS in patients with DKA

A multifactorial logistic regression model was constructed using the six predictor variables selected as independent variables using LASSO regression analysis (Fig. 2). The results revealed OASIS, GCS, AKI stage, vasoactive agents, and myocardial infarction as the risk factors for ICU LOS prolongation in patients with DKA (*P* < 0.05) (Table 2). A nomogram predicting the individual probability of prolonged ICU LOS in patients with DKA was constructed using the predictor variables. The nomogram was used to score the corresponding values of each variable, and subsequently, the scores of all variables were summed to obtain the total score. A vertical line was drawn downward according to the total score to indicate the estimated probability of prolonged ICU LOS in patients with DKA (Fig. 3).

**Table 2:** The result of Multivariate logistic analysis based on LASSO regression result

Variables	Multivariate logistic analysis		
	β	OR (95%CI)	*P*
GCS	-0.19	0.83 (0.72–0.95)	0.005
SOFA	0.133	1.14 (1.00-1.31)	0.057
OASIS	0.053	1.05 (1.01–1.10)	0.020
Myocardial infarct	0.910	2.48 (1.32–4.69)	0.005
Vasoactive agent	0.911	2.49 (1.05–5.92)	0.048
AKI Stage II-III	1.011	2.75 (1.53–4.59)	0.001

*Abbreviations* LASSO Least absolute shrinkage and selection operator, OR odds ratio, GCS Glasgow coma scale, SOFA Sequential organ failure assessment, AKI Acute kidney injury

### Discriminatory ability of the nomogram

**Fig. 4:**
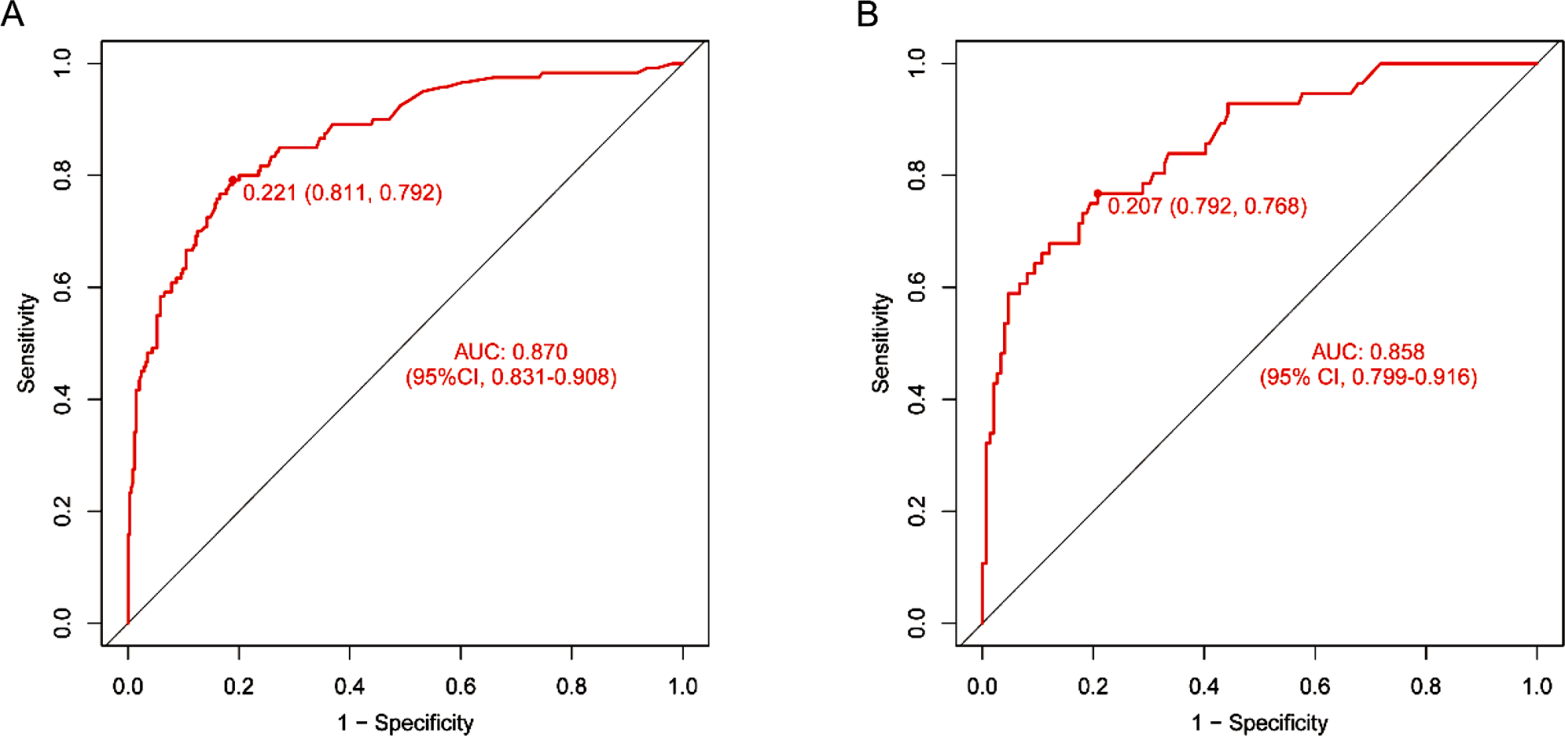
ROC curve of the nomogram prediction model. (**A**) training cohort, (**B**) validation cohort

The discriminatory ability of the nomogram was assessed by calculating the AUC and plotting the ROC curve for the predictive model. The AUC for the training cohort was 0.870 (95% confidence interval [CI], 0.831–0.908), with an optimal cut-off value of 0.221. In the validation cohort, the AUC was 0.858 (95% CI, 0.799–0.916) with an optimal cut-off value of 0.207. The results showed a relatively positive AUC in both cohorts, indicating that the nomogram prediction model has a good discriminatory ability (Fig. 4).

### Accuracy of the nomogram

**Fig. 5:**
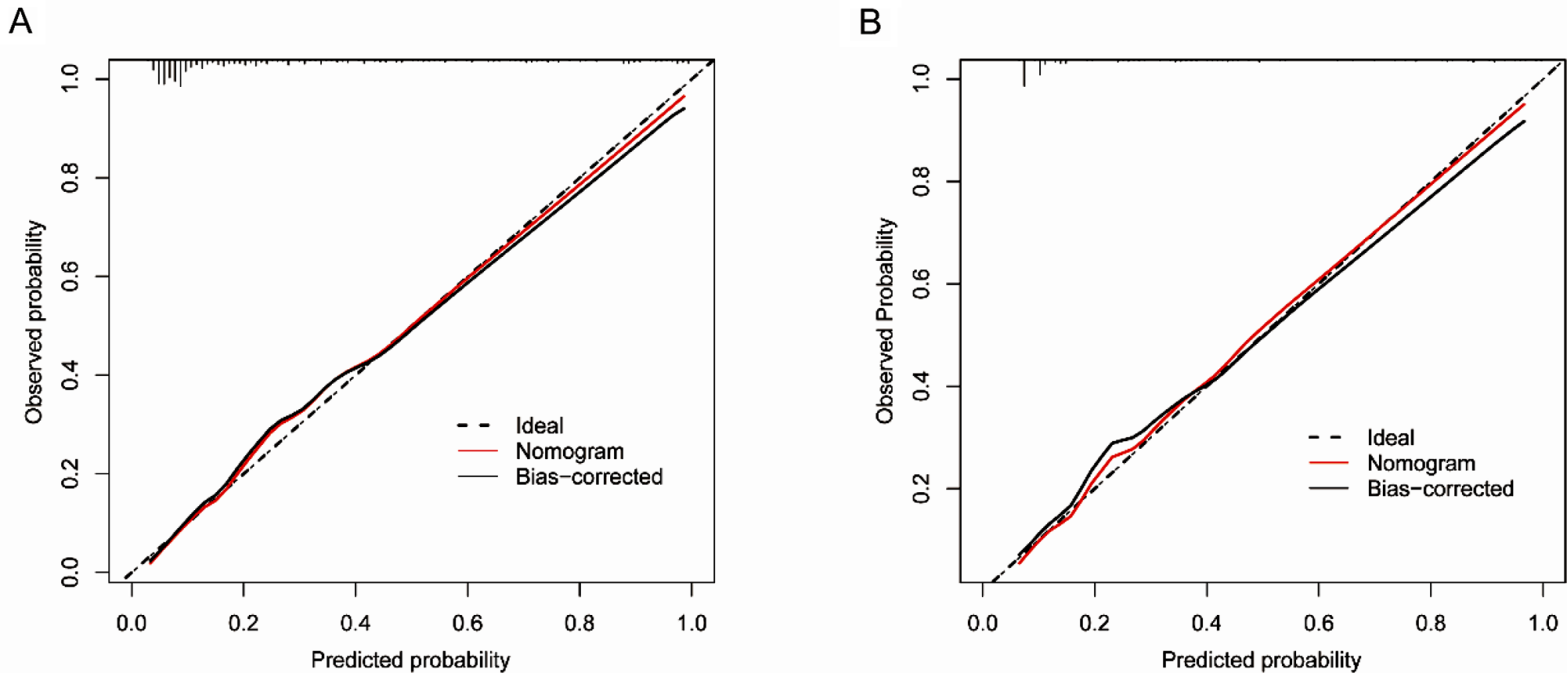
Calibration curve of the nomogram prediction model. (**A**) training cohort, (**B**) validation cohort

The Hosmer–Lemeshow test showed a good fit (*P* = 0.715 for the training cohort and *P* = 0.373 for the validation cohort), indicating that the predicted probability of the nomogram was consistent with the actual probability, demonstrating good calibration. In addition, calibration curves for both the training and validation cohorts showed moderate agreement, and the nomogram had a good calibration ability (Fig. 5).

### Clinical usefulness of the nomogram

**Fig. 6:**
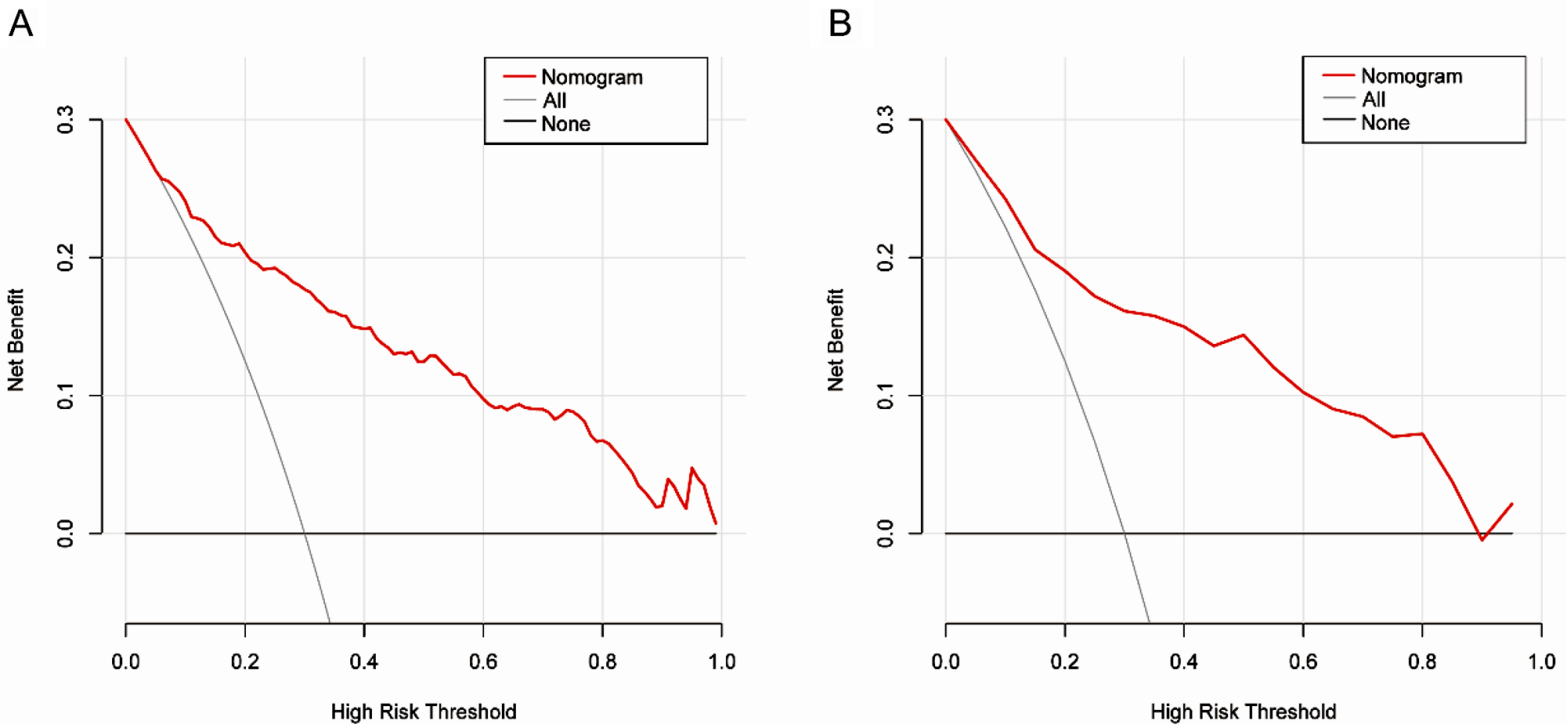
Decision curve analysis of the nomogram prediction model. (**A**) training cohort, (**B**) validation cohort

The clinical usefulness of the nomogram prediction model was assessed using DCA. The DCA for the nomogram was conducted in both the training and validation cohorts. The horizontal axis, indicating no one received the intervention, resulted in a net benefit of zero. The oblique line represents a scenario where all participants received the intervention. In the training cohort, predicted probability thresholds were set at 5–85%, with a net benefit ranging from 4 to 27%. In the validation cohort, predicted probability thresholds were set at 4–99%, with a net benefit ranging from 1 to 27%. Within this range, the nomogram’s net benefit was significantly higher than that of the two extreme cases, regardless of whether patients received clinical intervention (Fig. 6).

## Discussion

We constructed a nomogram based on the MIMIC-IV database to predict the risk of prolonged ICU LOS in patients with DKA. The nomogram robustness was further enhanced by screening for multiple factors using LASSO regression to avoid covariance and overfitting [12]. Subsequently, multifactorial logistic regression analysis was performed on the selected indicators. Ultimately, five key indicators, namely OASIS, GCS, AKI stage, vasoactive agents, and myocardial infarction were identified as predictors in this model. During model validation, the AUC of our nomogram was determined to be 0.870 and 0.858 in the training and validation cohorts, respectively, indicating a satisfactory predictive performance. Calibration plots demonstrated a satisfactory agreement between the actual and predicted values. Furthermore, the nomogram demonstrated good clinical utility through DCA. The nomogram developed in this study predicts the possibility of prolonged ICU LOS in patients with DKA based on medical history information, clinical investigations, and medications. Besides, it provides valuable clinical references for developing strategies to prevent and control prolonged ICU LOS in patients with DKA.

The OASIS score consists of 10 easily accessible basic parameters primarily used to assess the prognosis of critically injured patients [13]. In line with our findings, patients with DKA with high OASIS scores had longer ICU stays, suggesting that prolonged hospitalisation may be associated with poor prognosis.

Changes in the level of consciousness are important clinical symptoms and criteria for evaluating disease severity in patients with DKA. Cerebral oedema can occur in patients with DKA due to the combined effects of various factors, such as severe water loss, circulatory disorders, increased osmotic pressure, and cerebral cell hypoxia, causing central nervous system dysfunction and varying degrees of impaired consciousness [14, 15]. Fluid resuscitation is the initial step in relieving circulatory disturbances in patients with DKA. However, excessive fluid resuscitation can exacerbate cerebral oedema, thus titration of fluid resuscitation is crucial [16, 17]. Additionally, hypertonic therapy helps in transferring intracranial water into the bloodstream, ameliorating cerebral oedema with minimal impact on neurological outcomes [18]. Finally, balanced oxygen therapy proves effective in improving neurological outcomes by addressing ischemia and hypoxia in cerebral oedematous tissues thereby suppressing neuroinflammation [19]. Therefore, titration of fluid resuscitation, hypertonic therapy, and balanced oxygen therapy are essential to avoid cerebral oedema. GCS is one of the most commonly used clinical tools for assessing consciousness. Not incidentally, GCS was found to be a protective factor for prolonged ICU LOS in patients with DKA in our study (odds ratio: 0.83 (0.72–0.95), *P* = 0.005). Patients with a lower GCS indicate critical disease progression and tend to require longer ICU treatment duration.

We also examined the effect of the primary disease on ICU LOS in patients with DKA and found that myocardial infarction was an independent risk factor for prolonged ICU LOS. Issa et al. [20] found that myocardial infarction and the incidence of DKA are closely linked, with uncontrolled hyperglycaemia leading to elevated blood catecholamine levels, further exacerbating oxidative stress and causing endothelial and microvascular dysfunction [21]. Simultaneously, patients with myocardial infarction often experience hemodynamic and hormonal disturbances, further contributing to the development of DKA in patients with diabetes [22]. DKA and myocardial infarction can mutually influence and trigger each other. Determining the sequence of occurrence between these conditions is often challenging. Therefore, physicians managing patients with DKA must be vigilant of the potential presence of myocardial infarction, as it could contribute to prolonged ICU LOS in these patients.

We found that patients with comorbidities of AKI stages II–III tended to experience longer ICU LOS. AKI is a common complication in patients with DKA with high mortality and morbidity rates, especially in critically ill patients [23]. Renal ischaemia-reperfusion injury is a common cause of AKI, and permeability diuresis is a major risk factor for AKI in patients with DKA [1]. AKI is characterized by a sudden deterioration of renal function and a decrease in urine output, leading to disturbances in electrolyte and acid-base metabolism, volume overload, and damage to other organ systems as a result of these disturbances [24]. Thus, AKI progression is associated with DKA severity, further contributing to prolonged hospitalisation. In addition, similar to our findings, Fan et al. [25] found that a lower GCS score was also an independent risk factor for inducing AKI in patients with DKA, further proving that GCS is an important indicator in patients with DKA. Consequently, it underscores the need for heightened clinical vigilance toward changes in patient’s consciousness and the imperative for timely intervention during the early stages of disease progression.

Our study also found that the use of vasoactive agents was an independent risk factor for prolonged ICU LOS in patients with DKA, consistent with findings in other diseases [26]. The use of vasoactive agents suggests a state of hypoperfusion and hemodynamic instability [27], requiring prolonged ICU monitoring compared to patients not using such agents, ultimately resulting in prolonged ICU LOS.

To our knowledge, this is the first risk prediction model for prolonged ICU LOS in patients with DKA. This distinguishes our study from previous research [7, 10] that primarily focused on identifying factors influencing LOS in patients with DKA. In this study we simultaneously screened multiple variables using LASSO, enhancing the precision of the final inclusion in the nomogram. The five indicators included in our nomogram are relatively simple, easily accessible for critical care physicians and nurses, and can assist clinicians in making timely decisions and targeted interventions.

This study has some limitations. Firstly, this was a single-centre retrospective study, with a relatively small sample size, leading to potential selection bias and a less representative sample. Although the stability of our nomogram was tested by internal validation, further external validation across broader demographic groups is warranted based on our data. Secondly, owing to > 20% missing data in the database, our study did not include several potentially important factors, including glycated haemoglobin (HbA1c), glycaemic lability index [28, 29], albumin nutritional scores, urinary ketones, and causative factors for ketoacidosis. Thirdly, this study was limited by the current availability of databases. Although we found that DKA stage, hypoglycaemia, and mechanical ventilation may prolong ICU LOS in patients with DKA, these factors were not included in the multifactorial regression analyses, given the insufficient sample size and feasibility of predictive scores. These factors must be validated in future studies incorporating a larger number of centres. Finally, data were collected from patient case records. The accuracy of these records is important for model construction, which we cannot ascertain. Therefore, prospective cohorts should be included in subsequent studies to validate the stability of the model.

## Conclusion

The nomogram prediction model, constructed based on the five independent risk factors identified in this study, demonstrated good predictive efficacy in assessing the risk of prolonged ICU LOS in patients with DKA. After calibration to ensure reliable predictive accuracy, the model exhibited good clinical utility, as evidenced by DCA analysis. This can aid both patients and clinicians in determining prognosis and making informed clinical decisions. However, the recognized limitations underscore the need for ongoing research to explore additional influential factors, including HbA1c and glycaemic liability index. A comprehensive understanding of these variables will contribute to refining predictive modes and enhancing their effectiveness in guiding clinical decisions for optimal patient outcomes.

### Electronic supplementary material

Below is the link to the electronic supplementary material.

Supplementary Material 1: The missing rates for all excluded variables

## Data Availability

The data that support the findings of this study are available from the corresponding author upon reasonable request. Some data may not be made available because of privacy or ethical restrictions.

## Abbreviations

DKA: Diabetic ketoacidosis
HHS: Hyperosmolar hyperglycemia state
DM: Diabetes mellitus
T1DM: Type 1 diabetes mellitus
T2DM: Type 2 diabetes mellitus
AKI: Acute kidney injury
LOS: Length of stay
ICU: Intensive care unit
MIMIC-IV: Medical Information Mart for Intensive Care IV
LASSO: Least absolute shrinkage and selection operator
ROC: Receiver operating characteristic
AUC: Area under the curve
DCA: Decision curve analysis
GCS: Glasgow coma scale
SOFA: Sequential organ failure assessment
CCI: Charlson comorbidity index
LODS: Logistic organ dysfunction system
OASIS: Oxford acute severity of illness score
SAPSII: Simplified acute physiology score II
APSIII: Acute physiology score
BMI: Body mass index
RR: Respiratory rate
HR: Heart rate
MAP: Mean arterial pressure
UV: Urine volume
WBC: White blood cell
RDW: Red blood cell distribution width
AG: Anion gap
PH: Potential hydrogen
BUN: Blood urea nitrogen
POP: Plasma osmotic pressure
HbA1c: Glycated haemoglobin
CRRT: Continuous renal replacement therapy

## References

[1] Dhatariya KK, Glaser NS, Codner E, Umpierrez GE (2020). Diabetic ketoacidosis. Nat Rev Dis Primers.

[2] Umpierrez G, Korytkowski M (2016). Diabetic emergencies - ketoacidosis, hyperglycaemic hyperosmolar state and hypoglycaemia. Nat Rev Endocrinol.

[3] Benoit SR, Zhang Y, Geiss LS, Gregg EW, Albright A (2018). Trends in Diabetic Ketoacidosis hospitalizations and In-Hospital mortality - United States, 2000–2014. MMWR Morb Mortal Wkly Rep.

[4] Vellanki P, Umpierrez GE (2018). Increasing hospitalizations for DKA: a need for Prevention Programs. Diabetes Care.

[5] Virdi N, Poon Y, Abaniel R, Bergenstal RM (2023). Prevalence, cost, and Burden of Diabetic Ketoacidosis. Diabetes Technol Ther.

[6] Venkatesh B, Pilcher D, Prins J, Bellomo R, Morgan TJ, Bailey M (2015). Incidence and outcome of adults with diabetic ketoacidosis admitted to ICUs in Australia and New Zealand. Crit Care.

[7] Ata F, Khan AA, Khamees I, Iqbal P, Yousaf Z, Mohammed BZM, Aboshdid R, Marzouk SKK, Barjas H, Khalid M (2023). Clinical and biochemical determinants of length of stay, readmission and recurrence in patients admitted with diabetic ketoacidosis. Ann Med.

[8] Gosmanov AR, Gosmanova EO, Dillard-Cannon E (2014). Management of adult diabetic ketoacidosis. Diabetes Metab Syndr Obes.

[9] Azevedo LC, Choi H, Simmonds K, Davidow J, Bagshaw SM (2014). Incidence and long-term outcomes of critically ill adult patients with moderate-to-severe diabetic ketoacidosis: retrospective matched cohort study. J Crit Care.

[10] Freire AX, Umpierrez GE, Afessa B, Latif KA, Bridges L, Kitabchi AE (2002). Predictors of intensive care unit and hospital length of stay in diabetic ketoacidosis. J Crit Care.

[11] Johnson AEW, Bulgarelli L, Shen L, Gayles A, Shammout A, Horng S, Pollard TJ, Hao S, Moody B, Gow B (2023). MIMIC-IV, a freely accessible electronic health record dataset. Sci Data.

[12] Tibshirani R (1996). Regression shrinkage and selection via the lasso. J Royal Stat Soc Ser B: Stat Methodol.

[13] Johnson AE, Kramer AA, Clifford GD (2013). A new severity of illness scale using a subset of Acute Physiology and Chronic Health evaluation data elements shows comparable predictive accuracy. Crit Care Med.

[14] Siregar NN, Soewondo P, Subekti I, Muhadi M (2018). Seventy-two hour mortality prediction model in patients with Diabetic Ketoacidosis: a retrospective cohort study. J ASEAN Fed Endocr Soc.

[15] Obi MF, Namireddy V, Sharma M, Cho HJ, Udoyeh C, Morón Mercado LC, Htut Hann H (2023). An unfortunate Miss of undiagnosed arterial ischemic stroke (AIS) in the setting of Diabetic Ketoacidosis in an adult: a Case Report. Cureus.

[16] Sanfilippo F, La Via L, Dezio V, Amelio P, Genoese G, Franchi F, Messina A, Robba C, Noto A (2023). Inferior vena cava distensibility from subcostal and trans-hepatic imaging using both M-mode or artificial intelligence: a prospective study on mechanically ventilated patients. Intensive Care Med Exp.

[17] La Via L, Vasile F, Perna F, Zawadka M (2024). Prediction of fluid responsiveness in critical care: current evidence and future perspective. Trends Anaesth Crit Care.

[18] Cook AM, Morgan Jones G, Hawryluk GWJ, Mailloux P, McLaughlin D, Papangelou A, Samuel S, Tokumaru S, Venkatasubramanian C, Zacko C (2020). Guidelines for the Acute treatment of cerebral edema in Neurocritical Care patients. Neurocrit Care.

[19] Snyder B, Simone SM, Giovannetti T, Floyd TF (2021). Cerebral hypoxia: its role in Age-Related Chronic and Acute Cognitive Dysfunction. Anesth Analg.

[20] Issa M, Alqahtani F, Berzingi C, Al-Hajji M, Busu T, Alkhouli M (2018). Impact of acute diabetes decompensation on outcomes of diabetic patients admitted with ST-elevation myocardial infarction. Diabetol Metab Syndr.

[21] Esposito K, Nappo F, Marfella R, Giugliano G, Giugliano F, Ciotola M, Quagliaro L, Ceriello A, Giugliano D (2002). Inflammatory cytokine concentrations are acutely increased by hyperglycemia in humans: role of oxidative stress. Circulation.

[22] Marfella R, Verrazzo G, Acampora R, La Marca C, Giunta R, Lucarelli C, Paolisso G, Ceriello A, Giugliano D (1995). Glutathione reverses systemic hemodynamic changes induced by acute hyperglycemia in healthy subjects. Am J Physiol.

[23] Hoste EA, Bagshaw SM, Bellomo R, Cely CM, Colman R, Cruz DN, Edipidis K, Forni LG, Gomersall CD, Govil D (2015). Epidemiology of acute kidney injury in critically ill patients: the multinational AKI-EPI study. Intensive Care Med.

[24] Clec’h C, Darmon M, Lautrette A, Chemouni F, Azoulay E, Schwebel C, Dumenil AS, Garrouste-Orgeas M, Goldgran-Toledano D, Cohen Y (2012). Efficacy of renal replacement therapy in critically ill patients: a propensity analysis. Crit Care.

[25] Fan T, Wang H, Wang J, Wang W, Guan H, Zhang C (2021). Nomogram to predict the risk of acute kidney injury in patients with diabetic ketoacidosis: an analysis of the MIMIC-III database. BMC Endocr Disord.

[26] Cheng H, Li J, Wei F, Yang X, Yuan S, Huang X, Zhou F, Lyu J (2023). A risk nomogram for predicting prolonged intensive care unit stays in patients with chronic obstructive pulmonary disease. Front Med (Lausanne).

[27] Deng Y, Liu S, Wang Z, Wang Y, Jiang Y, Liu B (2022). Explainable time-series deep learning models for the prediction of mortality, prolonged length of stay and 30-day readmission in intensive care patients. Front Med (Lausanne).

[28] Hanna M, Balintescu A, Glassford N, Lipcsey M, Eastwood G, Oldner A, Bellomo R, Mårtensson J (2021). Glycemic lability index and mortality in critically ill patients-A multicenter cohort study. Acta Anaesthesiol Scand.

[29] Balintescu A, Palmgren I, Lipcsey M, Oldner A, Larsson A, Cronhjort M, Lind M, Wernerman J, Mårtensson J (2021). Prevalence and impact of chronic dysglycemia in intensive care unit patients-A retrospective cohort study. Acta Anaesthesiol Scand.

