# Supplementary material for: Clinical nomogram prediction model to assess the risk of prolonged ICU length of stay in patients with diabetic ketoacidosis: a retrospective analysis based on the MIMIC-IV database
Source: BMC Anesthesiol. 2024 Feb 29;24:86. doi: 10.1186/s12871-024-02467-z (PMC10902986; doi:10.1186/s12871-024-02467-z)
Supplement: Supplementary file 1 — Supplementary Material 1: The missing rates for all excluded variables [file 12871_2024_2467_MOESM1_ESM.docx]

| Variables | Total | Missing | Missing rate |
| --- | --- | --- | --- |
| Height | 669 | 142 | 21.2% |
| ALT | 669 | 158 | 23.6% |
| AST | 669 | 157 | 23.5% |
| ALP | 669 | 207 | 30.1% |
| HbA1c | 669 | 212 | 31.7% |
| albumin | 669 | 176 | 26.3% |
| C-reactive protein | 669 | 649 | 97.0% |
| Urine ketone | 669 | 182 | 27.2% |
| NTproBNP | 669 | 616 | 92.1% |

Table S1. The missing rate of all excluded variables

**Abbreviations:** ALT Alanine aminotransferase, AST Aspartate aminotransferase, ALP alkaline phosphatase, HbA1c Glycated hemoglobin A1c, NTproBNP N-terminal pro-B-type natriuretic peptide.
